# Biological Activity of Bark Extracts from Northern Red Oak (*Quercus rubra* L.): An Antioxidant, Antimicrobial and Enzymatic Inhibitory Evaluation

**DOI:** 10.3390/plants11182357

**Published:** 2022-09-09

**Authors:** Corneliu Tanase, Alexandru Nicolescu, Adrian Nisca, Ruxandra Ștefănescu, Mihai Babotă, Anca Delia Mare, Cristina Nicoleta Ciurea, Adrian Man

**Affiliations:** 1 Department of Pharmaceutical Botany, Faculty of Pharmacy, “George Emil Palade” University of Medicine, Pharmacy, Sciences and Technology of Târgu Mures, 38 Gheorghe Marinescu Street, 540139 Targu Mures, Romania; 2 Research Center for Medicinal and Aromatic Plants, “George Emil Palade” University of Medicine, Pharmacy, Sciences and Technology of Târgu Mures, 38 Gheorghe Marinescu Street, 540139 Targu Mures, Romania; 3 Faculty of Pharmacy, “Iuliu Hațieganu” University of Medicine and Pharmacy, 8 Victor Babes Street, 400012 Cluj-Napoca, Romania; 4 Doctoral School of Medicine and Pharmacy, “George Emil Palade” University of Medicine, Pharmacy, Sciences and Technology of Târgu Mures, 38 Gheorghe Marinescu Street, 540139 Targu Mures, Romania; 5 Department of Pharmacognosy and Phytotherapy, Faculty of Pharmacy, “George Emil Palade” University of Medicine, Pharmacy, Sciences and Technology of Târgu Mures, 38 Gheorghe Marinescu Street, 540139 Targu Mures, Romania; 6 Department of Microbiology, Faculty of Medicine, “George Emil Palade” University of Medicine, Pharmacy, Sciences and Technology of Târgu Mures, 38 Gheorghe Marinescu Street, 540139 Targu Mures, Romania

**Keywords:** antibacterial, antidiabetic, antifungal, antioxidant, bark, polyphenols, red oak, *Quercus*, tannins

## Abstract

The northern red oak (*Quercus rubra* L.) is an ornamental oak species native to eastern America, being an invasive species in Europe, with increasing coverage. The aim of this work was to evaluate the biological potential of red oak bark extracts. Aqueous and ethanolic preparations were obtained by two extraction methods: ultrasonic-assisted extraction (UAE) and microwave assisted extraction (MAE). The total phenolic and tannin contents were measured using spectrophotometric methods. The antioxidant activity was evaluated by two complementary methods (DPPH and ABTS). Antimicrobial potential was tested against five bacteria and three *Candida* species, and the effect on biofilm formation and synergism with gentamicin was also evaluated. Finally, enzyme inhibitory properties were assessed for α-glucosidase, tyrosinase, and acetylcholinesterase. The results indicated a higher phenolic content for the extracts obtained through MAE, while UAE bark extracts were rich in tannins. All the extracts exhibited antioxidant, anti-glucosidase, and anti-tyrosinase activity, while the antibacterial potential was mostly observed for the MAE extracts, especially against *S. aureus*, *C. parapsilopsis*, and *C. krusei*; inhibition of biofilm formation was observed only for MRSA. These findings show that the red oak bark might be an important source of bioactive compounds with antioxidant and antimicrobial properties.

## 1. Introduction

The northern red oak (*Quercus rubra* L. syn. *Q. borealis F.* Michx.) is an ornamental oak species belonging to the Fagaceae family, being native to eastern America [1]. It was introduced to Europe in 1961 and currently covers wide areas in northern hemisphere [1,2], where is cultivated for wood; unfortunately, it has lower quality and durability in comparison to native oaks (*Q. robur, Q. petraea*) [3].

*Q. rubra* is considered one of the most invasive tree species in temperate European forests. Due to its specific characteristics (rapid acclimatization, high ecological amplitude, and high wood production), red oak is considered one of the most frequently planted trees in European forests [4,5]. Some data have indicated that red oak influence the native forest in a different way, by decreasing light availability or by modifying soil properties [6].

The literature shows that many plant species have been tested to obtain extracts with high content of bioactive compounds (tannins, flavonoids, triterpenoids, phytosterols, peptides, polysaccharides, saponins, alkaloids, etc.) [7,8,9]. Among these, oak trees are an important source of bioactive compounds that have been tested for their biological activities [8,10]. The large number of species of this genus has led to a great diversity of extracts that can be investigated in terms of their biological potential. Polyphenols are the main compounds identified in species belonging to *Quercus* genus, but there are also cited other studies that have identified triterpenoids or polysaccharides [8,11,12,13].

Many studies show significant biological activity for the extracts obtained from oak trees (wood, bark, leaves, and acorns). This biological activity refers especially to the antioxidant capacity, antimicrobial, antiproliferative, immunomodulatory, or hypoglycemic effect [8].

To our knowledge, analyzing the current state of research, it was found that *Q. rubra* has not been studied yet in terms of phytochemical content or potential biological effect. Considering this, the aim of this work was to evaluate the phytochemical content and biological potential of red oak bark extracts (ROBE). Thus, the bark extracts (aqueous/ethanolic) obtained by ultrasonic assisted extraction (UAE) and microwave-assisted extraction (MAE) were evaluated for their antioxidant, antibacterial, and antifungal activity and enzyme inhibitory potential. The effect on biofilm formation and synergism with gentamicin were also evaluated.

## 2. Results

### 2.1. Polyphenol and Tannin Contents

All the determinations were made after redissolution of the freeze-dried ROBE (10 mg/mL), considering the absorption of the samples after the calibration curve of the gallic acid (GAE). The results were expressed as mg GAE/g dw ROBE, being presented in Table 1. The ROBE-ME had a significantly higher level of total polyphenols, suggesting that the MAE assured a better recovery of phenolic compounds from *Q. rubra* bark. It was also found that the water–ethanol solvent mixture improved the extraction yield of polyphenols for both extraction methods. On the other hand, the higher amounts of tannins were quantified in the extracts obtained through UAE, being observed again that hydroethanolic solvent increased the extraction yield for the tannins contained in the analyzed matrix.

### 2.2. Antioxidant Activity

**Figure 1:**
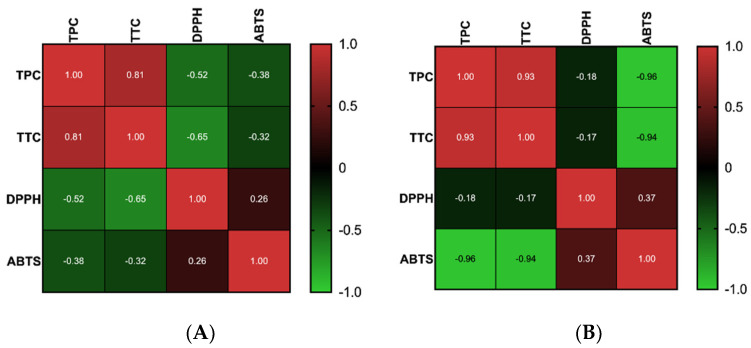
Pearson’s correlation coefficients among TPC, TTC, DPPH, and ABTS calculated for the extracts obtained by microwave-assisted extraction (**A**) and by ultrasound-assisted extraction (**B**).

Polyphenols, including tannins, are strong antioxidants due to their increased number of hydroxyl groups [14]. The antioxidant activity was measured using the ABTS and DPPH methods, the results being summarized in Table 1. The greatest antioxidant activity using the DPPH^•^ radical scavenging assay was recorded for ROBE-UE and ROBE-UA. For the ABTS^+•^ decolorization assay, the results indicated that hydroethanolic extracts have a statistically significant antioxidant activity compared with aqueous extracts. Pearson’s correlation between the main classes of metabolites and antioxidant activities, calculated for the two different methods of extraction, showed a very good correlation between TPC and TTC (Figure 1). The results also showed a good negative correlation between polyphenols and antioxidant activity determined by the DPPH method for the extracts obtained by MAE. For the extracts obtained through UAE, a very good negative correlation was obtained between the polyphenolic content and antioxidant activity measured by ABTS assay.

### 2.3. Antimicrobial Activity

The ROBE extracts were tested using the microdilution method. As can be observed in Table 2, ROBEs exerted antibacterial activity against all tested strains. Overall, the Gram-positive bacteria were more sensitive than the Gram-negative bacteria. For Gram-positive bacteria, the MIC values ranged from 0.3 to 5 mg/mL and MBC from 0.3 to 1.25 mg/mL, the highest sensitivity being proven for *S. aureus*. The MBC of ROBE-ME (0.3 mg/mL) was significantly higher than the other extracts. Among the Gram-negative bacteria, the most sensitive to the tested extracts was *K. pneumoniae* (MIC–0.6 mg/mL), while for *E. coli*, MIC and MBC values (greater than 5 mg/mL for all tested extracts) revealed the weakest antibacterial potential. It could be mentioned that these are the first results reported about the antibacterial activity of the extracts obtained from the red oak bark.

Antifungal potential was quantified on the basis of MIC values, presented in Table 3. Overall, a modest inhibition of fungal growth was observed. The extracts obtained through MAE were the most active; ROBE-MA showed a good activity against *C. parapsilopsis* and *C. krusei*, while ROBE-ME reached the minimal MIC against *C. krusei*. Both extracts obtained by UAE exhibited MIC valuer equal or higher than 5 mg/mL, which could be associated to a weak antifungal potential.

Further, the synergistic effect with gentamicin evaluated by the checkerboard method proved the ability of ROBE-ME (0.625–5 mg/mL) to synergically act with gentamicin (0.0626–8 mg/mL) against MRSA. Lower concentrations of ROBE-ME (0.002–0.313) proved no synergic effects with gentamicin against MRSA. In our experiment, we also evaluated the antibiofilm activity of the ROBEs for the bacteria where the highest antibacterial activity was observed (MRSA and *K. pneumoniae*). According to the obtained results (see Table 4), ROBE-UA, ROBE-UE, and ROBE-ME inhibited the formation of biofilms produced by MRSA. The inhibition effect was not observed for *K. pneumoniae* biofilms. Interestingly, except for ROBE-ME, biofilm inhibition varied in a non-dose-dependent manner.

### 2.4. Enzyme Inhibitory Activity

**Figure 2:**
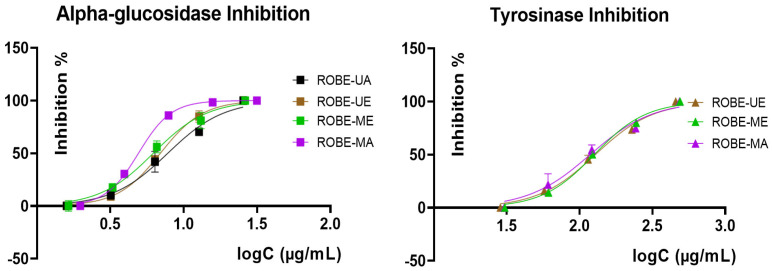
Graphs showing the dependence between the logC (of the concentration in terms of μg/mL) and the inhibition percentage for the ROBE samples in the case of α-glucosidase and tyrosinase inhibitory activity.

Regarding the enzyme inhibitory activity against some enzymes with relevance in human pathologies, namely, α-glucosidase, tyrosinase, and acetylcholinesterase, *Q. rubra* extracts were comparatively evaluated using the IC_50_ values (Table 5) extrapolated from 5 points inhibition curves obtained after the assessment of each enzyme (Figure 2; inhibition curves for acetylcholinesterase were not supplied as long as IC_50_ revealed a weak activity).

As can be observed, all samples exerted a strong anti-α-glucosidase potential, proven by the obtained IC_50_ values that were at least 10 times lower than those obtained for the positive control (acarbose, a standard inhibitor for this enzyme, used as a hypoglycemiant agent in the treatment of diabetes mellitus). Tyrosinase inhibition was moderate; except for ROBE-UA, all the extracts were able to act as moderate tyrosinase inhibitors, the best IC_50_ being obtained for the aqueous extract obtained through MAE (ROBE-ME). Lastly, acetylcholinesterase inhibition was the weakest for all tested *Q. rubra* bark extracts, with IC_50_ values revealing an insignificant activity in comparison with galantamine (positive control). It could be also observed that, in a similar manner as for the antibacterial potential, the extracts obtained via MAE were more active than those obtained through UAE, with this trend suggesting that microwave treatment improved the bioactive potential of *Q. rubra* bark herbal preparations.

## 3. Discussion

*Quercus* species barks are known for their long-term use as herbal medicine due to the presence of tannins and other phenolic compounds that are responsible for well-established antidiarrheal, hemostatic, anti-inflammatory, and antibacterial properties. European Pharmacopoeia [15,16] allows for the exclusive use of the bark collected from *Quercus robur* L., *Q. petraea* (Matt.) Liebl., and *Q. pubescens* Willd. but, in folk medicine, the product could be still collected also from other species, sometimes due to the accidental substitution or the low availability of the officinal ones in some areas [8,16,17]. However, during the last decades, the interest for the extensive valorization of this product increased; the bark is one of the main by-products resulting after the primary processing of *Quercus* species in the wood industry and, at the same time, was proven as a source of natural biomolecules with promising antibacterial or antihyperglycemic activity [18,19].

As we previously mentioned, there is lack of knowledge regarding phytochemical and bioactive characterization of *Q. rubra* bark, with our preliminary evaluation highlighting for the first time the potential use of this product as an alternative source of phenolic compounds, especially tannins. Moreover, this study emphasizes the importance of modern alternative extractive techniques as suitable methods for enhancing the recovery of phytochemicals from herbal matrices and improving the quality of the final extracts in terms of chemical and bioactive properties. This trend could be also observed in our results, especially in the evaluation of total phenolic and tannins contents. Overall, the microwave treatment increased the TPC values, while the extraction yields for tannins were improved by using ultrasounds and hydroalcoholic mixtures as solvents. It is well known that UAE induces specific processes during the extraction, namely, cell wall disruption and cavitation [20,21], allowing a better contact between intracellular environment and solvent, enhancing the partition of bioactive compounds between the extracted matrix and solvent. In the case of *Quercus* barks, tannins are usually found as brown or reddish intracellular accumulations in lignified tissues (xylem fibers, groups of parenchymatous sclereids, cork cells) [15] strongly impermeable for water, which prevents their proper extraction by using classic methods mainly based on partition law. Hence, the use of hydroethanolic solvent and UAE could be considered as part of a suitable strategy for the extraction of tannins from *Q. rubra* bark.

Quantitative distribution of polyphenols in ROBE indicates similar (303.4 mg GAE/g for bark of *Q. cerris var. cerris—*MeOH, 259 mg GAE/g for bark of *Q. cerris var. cerris—*H_2_O, 327.1 mg GAE/g for bark of *Quercus macranthera* subsp. *syspirensis*–H_2_O) or higher values (68–79 mg GAE/g for bark of *Q. robur*) than for the same product collected from other *Quercus* species, with a similar trend being shown for the tannin’s contents. The presence of these secondary metabolites could be correlated with the antioxidant, antibacterial, and enzyme inhibitory potentials of the extracts. As can be observed, ROBE-UE exerted the most antioxidant activity (both in DPPH and ABTS assays), which can be explained by the additive effect of tannins and the other polyphenols contained in this extract, with both types of phytochemicals being well-known as main antioxidant agents. Differences between the results obtained from the two antioxidant assays can be explained by the individual features of the two radicals: the ABTS^+•^ is suitable for both lipophilic and hydrophilic antioxidant systems, while DPPH^•^ is suitable only for the hydrophilic antioxidant systems. The results obtained for red oak bark extracts are similar with those ones previously reported by Santos et al. [22] who evaluated *Q. suber* cork extracts for their antioxidant potential using DPPH assay.

Antimicrobial properties of ROBEs were also influenced by the quantitative distribution of total tannins and other phenolic compounds (quantified as TPC) in their composition. Multiple mechanisms are involved in the antimicrobial activity of tannins, the most studied being the interaction with surface proteins, the damage of the activity of lipid bilayer membranes or cell walls, and the ability of interfere with some metallic ions (especially iron) that act as cofactors for key enzyme-dependent processes [23,24]. The most important antibacterial compounds belonging to the tannins group previously reported in *Quercus* bark extracts are gallic acid, ellagic acid, vescalagin, and castalagin [25]. On the basis of the obtained results, our findings suggest an additive antimicrobial effect between tannins and other polyphenol metabolites found in *Q. rubra* bark extracts; this hypothesis could be supported by the MIC and MBC values that were lower for the extracts prepared through MAE, which showed medium TPC and TTC values (interestingly, tannin-rich extracts such as ROBE-UE and ROBE-UA exerted a weaker antimicrobial potential). Moreover, in terms of antibacterial effect, ROBEs were more active against Gram-positive strains, which contrasts with the previous reports on the antibacterial activity of other *Quercus* species such as *Q. cerris* [18], *Q. leucotricophora* [26], *Q. crassifolia* [27], *Q. robur*, and *Q. accutissima* [28]. In a similar manner, extracts obtained through microwave treatment showed the best antifungal potential, which was comparable with that described in a previous study on *Quercus robur* bark extract on *C. albicans* using the agar diffusion method [29]. Antifungal activities of bark extracts from *Q. robur, Q. macrocarpa*, and *Q. acutissima* against *Aspergillus flavus*, *Penicillium funiculosum*, *P. ochrochloron*, and *C. albicans* were also proven by Elansary et al. [28] and correlated with the presence of ellagic acid, flavan-3-ols, and caffeic acid derivatives, the main constituents of the analyzed oak species barks.

Additionally, for the direct antibacterial activity, phytocomplexes obtained from various plant sources could interfere with pathogen microorganisms’ growth through other indirect mechanisms. The synergistic effect is defined as the interaction between two agents, where one agent enhances the effect of the other and together they act more effectively [30]; in particular, our study describes the synergistic antibacterial effect of ROBE in combination with gentamicin, which was previously proven for other bark extracts [30,31]. Today, the priorities in the medical field can be the reduction of antibiotic resistance and the search for new antimicrobial agents, capable of modulating bacterial virulence, such as adhesiveness and biofilm formation. Recent studies showed that the secondary metabolites of plants and especially polyphenols exert antibacterial and/or antibiofilm activities [32,33,34]. Chusri et al. [35] showed that at the MIC value, *Quercus infectoria* extract, significantly reduced MRSA biofilm formation, probably due to the effect of the extract on the hydrophobicity of the bacterial cell surface and cell wall that could confer anti-biofilm activity. Hence, our results are in line with previous findings regarding the ability of oak species (including *Q. rubra*) bark extracts to act as biofilm formation inhibitors.

Even that the extracts of *Quercus* species barks are generally recognized for a couple of bioactive potentials intensively exploited in phytotherapy (previously mentioned at the beginning of this section); recent research has been focused on reconsidering these products as valuable complementary agents in the treatment of some chronic diseases, including diabetes mellitus [17,36,37]. The antidiabetic effect of different *Quercus* species is mainly associated with the ability of their phytocomplexes or isolated compounds to inhibit the activity of two main enzymes involved in glucose metabolism, namely, *α*-glucosidase and *α*-amylase [37]. Sari et al. [25] proved recently the inhibitory potential of *Q. infectoria* bark against *α*-glucosidase, this effect being attributed to the presence of several tannins derivatives (i.e., (-)-8-chlorocatechin, 3-*O*-digalloyl-1,2,4,6-tetra-*O*-galloyl-*β*-D-glucopy-ranoside) as main constituents of the analyzed extracts. Furthermore, *Q. gilva* [38], *Q. suber* [39], and *Q. mongolica* [40] extracts were also tested for their anti-*α*-glucosidase potential, showing lower IC_50_ values than the positive control (acarbose). Various classes of phytocomponents were proven to interact with the enzymes responsible for glucose metabolism [41,42]; among them, tannins exhibit inhibitory properties via their ability to establish multiple hydrogen bonds and hydrophobic associations with proteic structures, inducing irreversible conformational changes and blocking enzyme catalytic sites. It must be also noticed that this mechanism can be influenced by the presence of other compounds in the extracts; thus, the stronger inhibitory activity of ROBEs against *α*-glucosidase could probably explained by the synergic additive of tannins and the other phenolic compounds present in these herbal preparations.

Higher tannin content of *Quercus* species bark could also explain its ability to act as a tyrosinase inhibitor [43]. This enzyme catalyzes oxidative transformations involved in formation of melanin, the main compound responsible for skin pigmentation. Several skin disorders are linked with defectuous melanogenesis, and hence the research for alternative tyrosinase inhibitors among natural sources is increasing [43]. Several *Quercus* species were previously evaluated for their potential ability to modulate tyrosinase activity: *Q. coccifera* bark extracts showed an in vitro inhibitory activity against tyrosinase comparable with kojic acid (a well-established tyrosinase inhibitor used as medicine in topical formulations) [25], while *Q. infectoria* showed a moderate activity (extracts exerted an inhibition rate of 59.3% for the enzyme at a concentration of 100 μg/mL) [44]. Hence, as well as for *α*-glucosidase, tyrosinase inhibitory potential of ROBEs was evaluated for the first time in this study, revealing the potential use of *Q. rubra* bark as a potential alternative source of phytochemicals with relevance in supportive therapy of diabetes mellitus or skin disorders related to hyperproduction of melanin. Moreover, our findings suggest that it could be established that there is an interdependence between the extraction procedure applied for the preparation of the extracts and their enzyme inhibitory potency, with it being observed that phytocomplexes obtained through microwave-assisted treatment showed the lowest IC_50_ values in the antienzyme assays.

## 4. Materials and Methods

### 4.1. Chemicals, Reagents, and Bacterial Strains

The chemicals used were ethanol (pro analysi), Na_2_CO_3_ decahydrate purchased from Reactivul SRL (Ramnicu Valcea, Romania), gallic acid monohydrate purchased from Sigma-Aldrich Chemie GmbH (Steinheim, Germany), DMSO (analytical grade, ACS reagent), and Folin–Ciocâlteu reagent purchased from Merck KGaA (Darmstadt, Germany). The protocols used for the assessment of total tannin content and antioxidant activity additionally required 2,2-diphenyl-1-picrylhydrazyl (DPPH), hide powder, and pyrogallol, all acquired from Sigma-Aldrich Chemie GmbH (Steinheim, Germany) and 2,2′-azino-bis(3-ethylbenzothiazoline-6-sulfonic acid) (ABTS) tablets purchased from Roche Diagnostics (GmbH, Göttingen, Germany).

The antimicrobial activity was evaluated against five bacterial strains (*Staphylococcus aureus* ATCC 25923, methicillin-resistant *Staphylococcus aureus* (MRSA) ATCC 43300, *Escherichia coli* ATCC 25922, *Klebsiella pneumoniae* ATCC 13883, *Pseudomonas aeruginosa* ATCC 27853) and three *Candida* species, namely, *C. albicans* ATCC 90028, *C. parapsilosis* ATCC 22019, and *C. krusei* 6258, all of them provided by the Microbiology Department of the George Emil Palade University of Medicine, Pharmacy, Sciences, and Technology from Târgu-Mureș.

The reagents used for the determination of enzyme inhibitory activity, namely, α-glucosidase (from *Saccharomyces cerevisiae*), p-nitrophenyl α-D-glucopyranoside (PNPG), tyrosinase (from mushroom), L-DOPA, acetylcholinesterase (from *Electrophorus electricus*), 5,5′-dithiobis(2-nitrobenzoic acid) (DTNB), acetylthiocholine iodide (ATCI), acarbose, kojic acid, galantamine, potassium phosphate dibasic (K_2_HPO_4_), potassium phosphate monobasic (KH_2_PO_4_), and tris-HCL were purchased from Sigma-Aldrich, Merck (Darmstadt, Germany).

### 4.2. Plant Sample

The norther red oak (*Quercus rubra* L.) bark was provided from Bistrița-Năsăud County (47°22′17″ N, 24°16′32″ E), Romania, in June 2020. The age of the trees was 20–30 years. The bark was collected by using the itinerary method, performed from the stems and splintered manually from the tree trunk 1 m from the ground. The species was identified and authenticated by Dr. Corneliu Tanase from the Department of Pharmaceutical Botany, George Emil Palade University of Medicine, Pharmacy, Sciences, and Technology from Târgu-Mureș. The bark was dried in an oven at 50 °C for 24 h. The dried material was then milled using a Pulverisette 15 cutting mill (Fritsch GmbH, Idar-Oberstein, Germany).

### 4.3. Extraction

The ultrasound-assisted extraction (UAE) was performed at 40 kHz ultrasonic power. Thus, 2.5 g bark was placed into a volumetric flask with 100 mL water (100%) or ethanol/water (50:50 *v*/*v*) solvent. Extraction mix was ultrasonicated for 15 min, under a constant temperature 70 °C monitored during all the extraction times, in order to assure a proper and reproductible recovery of bioactive compounds from the extracted matrix [45].

For the microwave-assisted extraction (MAE), 10 g of red oak bark was placed into the microwave extractor vessel with 200 mL of water or ethanol/water (70:30 *v*/*v*). The extractions were performed in an Ethos X Advanced microwave extractor (Milestone, Sorisole, Italy). The extraction conditions for the aqueous extracts were 30 min at 850 W and for the hydroalcoholic extracts 18 min at 650 W [18]. The obtained extracts were centrifuged, concentrated, and freeze dried. Thus, the extract samples were (1) red oak bark extract obtained by ultrasound-assisted extraction with water solvent (ROBE-UA); (2) red oak bark extract obtained by ultrasound-assisted extraction with ethanol solvent (ROBE-UE); (3) red oak bark extract obtained by microwave-assisted extraction with water solvent (ROBE-MA); (4) red oak bark extract obtained by microwave-assisted extraction with ethanol solvent (ROBE-ME).

### 4.4. Quantification of Total Phenolics

The total polyphenolic content (TPC) of the ROBE was assessed using the Folin–Ciocâlteu method, as previously described, with slight modifications [46].

The TPC was determined by adding 400 µL of Folin–Ciocâlteu reagent to 400 µL of diluted extract (10 mg/mL), and finally adding 3200 µL of 5% Na_2_CO_3_ solution, shaking the test tubes well and leaving them at room temperature in darkness for one hour. For each diluted extract, 3 replicates were performed. The absorbance of each replicate was measured at 750 nm with a Specord 200 Plus (Analytik Jena AG, Jena, Germany). The results were expressed as mg GAE/g dried bark.

### 4.5. Quantification of Tannins

Quantification of tannins was performed according to the hide powder assay described in European Pharmacopoeia 10.8 [47]. Absorbance was read at 760 nm using a Specord 200 Plus (Analytik Jena AG, Jena, Germany). The results were expressed as % pyrogallol/extract.

### 4.6. Antioxidant In Vitro Assays

#### 4.6.1. Determination of DPPH Radical Scavenging Activity

The assay was conducted according to a previously described method [18]. Briefly, the lyophilized extracts were redissolved (1 mg/mL) in the same solvent used for extraction. An aliquot of each extract was added in the first well of a microplate. Binary dilutions were prepared for each sample, and after homogenization, 200 µL DPPH 0.1 mM was added in each well. The absorbance was read at 517 nm for each mixture, after 30 min, using a microplate reader (Epoch, BioTek, Winooski, VT, USA).

Inhibition capacity was calculated using Formula (1):

(1) $$\mathrm{inhibition}\;(\%)=\frac{A_{c}-A_{s}}{A_{c}}\times 100$$

where *A_c_* is the absorbance of control (Trolox), and *A_s_* is the absorbance of sample.

Half maximal inhibitory concentration (IC_50_) was calculated using a dose–response curve and was expressed as µg/mL.

#### 4.6.2. Determination of ABTS Free Radical-Scavenging Activity

The ABTS^•+^ decolorization assay was performed according to the method described by Laczkó-Zöld et al., with slight modifications [48]. The bioassay was performed in a 96-well microplate, where each well contained 50 µL extract (1–0.625 mg/mL) or water as control and 200 µL ABTS^•+^ solution. The mixture was allowed to react for 6 min, and the absorbance was measured at 734 nm using a microplate reader (Epoch, BioTek, Winooski, VT, USA). Inhibition capacity and IC_50_ were calculated as described above (see Equation (1)).

### 4.7. Assay of the Antimicrobial Activity

#### 4.7.1. Microdilution Method

The antibacterial potential of the ROBE was evaluated using a previously reported microdilution method that was slightly modified [49]; *Staphylococcus aureus* ATCC 25923, MRSA ATCC 43300, *Escherichia coli* ATCC 25922, *Klebsiella pneumoniae* ATCC 13883, and *Pseudomonas aeruginosa* ATCC 27853 were the bacterial microorganisms used as reference strains. ROBEs were dissolved in purified water containing 5% DMSO at a concentration of 10 mg/mL and filter sterilized (0.2 µm PES syringe filters, Whatman Puradisc 25 mm); 200 µL from each sample were supposed to serial dilutions in a 96-well plate, being further mixed with 100 μL of bacterial suspension of each strain (previously prepared using 10 µL of 0.5 McFarland inoculum and 9990 µL of Muller–Hinton broth 2×). After incubation at 37 °C for 24 h, minimum inhibitory concentration (MICs) (defined as the lowest concentration that was able to completely inhibit the bacterial growth) was measured for each extract using optical evaluation. The minimum bactericidal concentration (MBC) was assessed by subculturing 5 µL from 3 consecutive wells (from the MIC-well and from the two more concentrated dilutions) on blood-agar plates, followed by incubation at 37 °C for 24 h. The dilution, which did not allow any bacterial growth on blood agar, was considered the MBC.

In a similar way, antifungal potential was tested against *Candida albicans* ATCC 90028, *Candida parapsilosis* ATCC 22019, and *Candida krusei* ATCC 6258. First, fungal inoculums were obtained from each fungal strain by mixing 0.5 McFarland and 9 mL Gibco Roswell Park Memorial Institute medium (RPMI) buffered with 3-(n-morpholino)propanesulfonic acid (MOPS) and supplemented with 2% glucose. Samples were supposed to serial dilutions in a 96-wells microplate and mixed with 100 μL of inoculum, with MIC values being appreciated using visual evaluation.

#### 4.7.2. The Checkerboard Method–Gentamicin Synergy Test

The checkerboard method was used to study the synergy between gentamicin and the ROBE, which was proven to have antibacterial activity. Successive dilutions of the ROBE and gentamicin were prepared in a microtiter plate. The dilution was performed in distilled water directly on the microtiter plate as in the case of MIC assay; gentamicin dilution was performed in Muller–Hinton medium 2×. The bacterial suspensions were created by mixing 20 µL inoculum 0.5 McFarland with 9980 µL Muller–Hinton 2×. According to the design of the plate, the final volume of each well was 200 µL (100 µL red oak extracts + 50 µL gentamicin solution + 50 µL bacterial suspension). The negative control was considered in well A1 (maximum amounts of gentamicin and test substance), and the positive control in well H12 (minimum amounts of gentamicin and test substance). The MIC in the case of gentamicin was the maximum dilution of gentamicin in the presence of the minimum test substance at which no bacterial growth was observed, and the MIC of the test substance was the maximum dilution of the test substance in the presence of the minimum concentration of gentamicin in which no bacterial growth was observed. For the wells in which no bacterial growth was observed, the fractional inhibitory concentration (FIC) was calculated according to the following formula (see Equation (2)):

FIC = FIC1 + FIC2(2)

FIC1—gentamicin concentration in the analyzed well/MIC gentamicin.

FIC2—concentration of ROBE in test well/MIC of ROBE.

In the wells where the FIC value ≤ 0.5, this indicates a synergistic effect; FIC = 0.5–2 means the effect is indifferent; FIC > 2 shows an antagonistic effect.

#### 4.7.3. The Effect on the Formation of Bacterial Biofilm

To evaluate the effect of ROBE on the bacterial biofilm formation by MRSA and *K. pneumoniae*, the following dilutions of the extracts were tested: 3%, 1.5%, 0.75% (these concentrations were chosen according to MIC results obtained from the assay described in Section 4.7.1). The tested bacterial strains are able to produce biofilm in the presented conditions and were assessed in the positive control wells (bacteria + culture medium alone). The biofilm indexes of the bacteria + culture medium + ROBE were calculated in reference to this control well.

Thus, 0.5 McFarland bacterial inoculum was performed in sterile saline, the bacterial suspension being then prepared from 10 µL inoculum + 9990 µL RPMI medium. The dilutions of extracts (3%, 1.5%, 0.75%) were performed in RPMI (low-nutritional environment). In the microtiter plate, the final volume of the well was 200 µL (100 µL bacterial suspension + 100 µL test substance diluted in RPMI). The growth controls (bacterial suspension + RPMI) and negative control (RPMI, RPMI + test substance) were performed. The plates were incubated at 37 °C for 18–24 h, and then the excess of RPMI was removed and the plates were washed by immersion in sterile water twice to remove non-adherent cells. A total of 200 µL of 0.1% crystal-violet aqueous solution was pipetted into each well, and the plates were left for 15 min at room temperature. The excess crystal-violet was removed by immersion 3 times in sterile water and the plates were left at room temperature until dry. After drying, in each well, 200 µL of 30% acetic acid was pipetted, and the plates were left at room temperature for 15 min to solubilize the purple crystal adhered to the biofilm. The absorbance of each well was read at 590 nm using the microplate reader. The biofilm inhibition was calculated as an index according to the following formula (see Equation (3)):

BII = (TS − C)/CG(3)

BII—biofilm inhibition index; TS—absorbance of the studied well; C—absorbance of control; CG—absorbance of control in growth. Index value below 0.75—inhibition, over 1.25—stimulation (values between 0.75 and 1.25 were attributed to the chance error).

The transformation into biofilm inhibition percentages was performed using Equation (4):

% biofilm inhibition = (100 × BII) − 100(4)

percentage value between −100% and −75% was considered inhibition, and a value over +25% was considered stimulation (values between −25% and +25% were attributed to chance error).

### 4.8. Enzyme Inhibitory Activity

ROBE samples were tested for their in vitro inhibitory potential against α-glucosidase, tyrosinase, and acetylcholinesterase. In the α-glucosidase inhibition assay, 50 μL of the diluted extract (serial dilutions between 100 and 6.25 μg/mL) with different concentrations were mixed with 50 μL of enzyme solution in phosphate buffer (pH 6.8) and 50 μL of 10 mM PNPG in phosphate buffer. After the incubation (37 °C for 15 min), the absorbance of each sample was recorded at 400 nm using as positive control acarbose [50].

Tyrosinase inhibition was assessed by mixing 25 μL of diluted extract (serial dilutions between 2.5 and 0.156 mg/mL), 40 μL of enzyme (10 U/mL), and 100 μL of phosphate buffer (pH 6.8). After 15 min of incubation at room temperature, the reaction mixture was completed by adding 40 μL of L-DOPA (2.5 mM in phosphate buffer) and re-incubated for 10 min. Finally, the absorbance was read at 492 nm using kojic acid as positive control [20,51].

Inhibition of acetylcholinesterase was measured through a previously modified version of Ellman’s method [51,52]. The reaction was initiated by mixing diluted (serial dilutions between 4 and 0.25 mg/mL) extract (25 μL) with Tris-HCl buffer (50 μL; 50 mM, pH 8.0), DTNB solution in Tris-HCl buffer (125 μL; 0.9 mM), and enzyme solution (25 μL; 0.078 U/mL), followed by 15 min incubation in a dark place at room temperature. Further ATCI solution (25 μL; 4.5 mM in Tris-HCl buffer) was added, with the samples then being re-incubated for 10 min. Finally, the absorbance of each sample was recorded at 405 nm using galantamine as a positive control.

The percentages of inhibition (*I*, in %) for enzyme inhibition assay were calculated using the following formula (see Equation (5)):

(5) $$I(\%)=\frac{A_{c}-A_{s}}{A_{c}}\times 100$$

where *A_c_* is the absorbance of control solution (galantamine) and *A_s_* is the absorbance of sample.

The results were expressed as IC_50_ (the concentration of the sample that was able to inhibit 50% of the enzyme, in μg/mL), considering the dilution in the 96 wells for each sample and not the original concentration of the re-solubilized sample. The IC_50_ was calculated using the normalized logarithmic curve for the determined percentages of inhibition, and the dependence between the logC and the I (%) was graphed using Prism 8 (GraphPad, San Diego, CA, USA).

## 5. Conclusions

Our present work emphasizes for the first time the bioactive potential of phenolic- and tannin-rich aqueous and hydroethanolic fractions obtained through microwave- and ultrasound-assisted extraction from the bark of *Q. rubra*, an invasive oak species commonly widespread in the northern hemisphere. Ultrasound treatment enhanced the recovery of tannins, while microwave-assisted extraction allowed us to obtain extracts with a higher total phenolic content. In a similar manner, the use of ethanol–water mixture improved the recovery of bioactive compounds from the analyzed matrix. In terms of bioactive potential, antioxidant, antibacterial, *α*-glucosidase, and tyrosinase inhibitory properties could be attributed to the important amounts of tannins found in the extracts or to a possible additive effect between these compounds and other phenolic constituents. However, these preliminary findings support the potential use of *Q. rubra* bark for the development of herbal products with anti-infectious or antidiabetic effects, encouraging at the same time further deepening of the evaluation of individual compounds responsible for the aforementioned bioactivities.

## Figures and Tables

**Table 1:** Total polyphenolic content (TPC), total tannin content (TTC), and antioxidant activity of ROBE.

Sample	TPC (mg GAE/g dw)	TTC (%)	IC50 DPPH (µg/mL)	IC50 ABTS (µg/mL)
ROBE-UA	203.58 ± 3.25 ^d^	37.96 ± 1.01 ^b^	3.54 ± 0.48 ^c,d^	7.41 ± 0.09 ^b^
ROBE-UE	226.79 ± 1.54 ^b^	44.75 ± 2.63 ^a^	3.32 ± 0.13 ^e^	6.07 ± 0.22 ^c^
ROBE-MA	216.47 ± 1.19 ^c^	24.67 ± 1.53 ^c^	6.91 ± 0.39 ^a^	8.28 ± 0.13 ^a^
ROBE-ME	321.08 ± 3.23 ^a^	37.82 ± 7.57 ^b^	6.24 ± 0.66 ^b^	7.89 ± 0.75 ^b^

Note: dw—dry weight. Different superscript letters (a–d) in the same column mean statistically significant differences at *p* < 0.05 and the same alphabetical superscript in a column indicates no statistically significant difference; ROBE-UA: red oak bark extract obtained by ultrasound-assisted extraction with water solvent; ROBE-UE: red oak bark extract obtained by ultrasound-assisted extraction with ethanol solvent; ROBE-MA: red oak bark extract obtained by microwave-assisted extraction with water solvent; ROBE-ME: red oak bark extract obtained by microwave-assisted extraction with ethanol solvent.

**Table 2:** Antibacterial activity (MIC and MBC in mg/mL) of the ROBEs.

	ROBE-UA		ROBE-UE		ROBE-MA		ROBE-ME	
Gram-positive bacteria								
	MIC	MBC	MIC	MBC	MIC	MBC	MIC	MBC
*S. aureus*	0.3	1.25	0.3	1.25	0.6	5	0.3	0.3
MRSA	1.25	1.25	1.25	1.25	1.25	1.25	0.6	0.6
Gram-negative bacteria								
*E. coli*	>5	>5	>5	>5	>5	>5	>5	>5
*K. pneumoniae*	0.6	2.5	0.6	5	0.6	0.6	1.25	5
*P. aeruginosa*	2.5	5	2.5	5	>5	>5	2.5	5

Note: MIC: minimum inhibitory concentration; MBC: minimum bactericidal concentration; MRSA: methicillin-resistant *Staphylococcus aureus*; ROBE-UA: red oak bark extract obtained by ultrasound-assisted extraction with water solvent; ROBE-UE: red oak bark extract obtained by ultrasound-assisted extraction with ethanol solvent; ROBE-MA: red oak bark extract obtained by microwave-assisted extraction with water solvent; ROBE-ME: red oak bark extract obtained by microwave-assisted extraction with ethanol solvent.

**Table 3:** Antifungal activity (MIC in mg/mL) of the ROBEs.

	*C. albicans*	*C. parapsilosis*	*C. krusei*
ROBE-UA	>5	5	5
ROBE-UE	>5	>5	5
ROBE-MA	>5	0.3	0.02
ROBE-ME	>5	5	2.5

Note: MIC: minimum inhibitory concentration; ROBE-UA: red oak bark extract obtained by ultrasound-assisted extraction with water solvent; ROBE-UE: red oak bark extract obtained by ultrasound-assisted extraction with ethanol solvent; ROBE-MA: red oak bark extract obtained by microwave-assisted extraction with water solvent; ROBE-ME: red oak bark extract obtained by microwave-assisted extraction with ethanol solvent.

**Table 4:** The effect of ROBE on bacterial biofilm formation.

		MRSA		*K. pneumoniae*	
Sample	Concentration (%)	BII (%)	SD	BII (%)	SD
ROBE-UA	3	−40.40	0.00	21.00	0.00
	1.5	−41.60	0.00	14.40	0.00
	0.75	−41.60	0.00	12.60	0.00
ROBE-UE	3	−40.40	0.00	10.80	0.01
	1.5	−41.30	0.00	15.60	0.00
	0.75	−44.50	0.00	10.80	0.00
ROBE-MA	3	11.53	0.60	19.72	0.06
	1.5	25.73	0.01	35.78	0.01
	0.75	3.49	0.05	77.53	0.09
ROBE-ME	3	−51.00	0.00	3.60	0.00
	1.5	−49.90	0.00	0.60	0.00
	0.75	−43.10	0.01	1.20	0.00

Note: MRSA: methicillin-resistant *Staphylococcus*; BII: biofilm inhibition index; SD: standard deviation; ROBE-UA: red oak bark extract obtained by ultrasound-assisted extraction with water solvent; ROBE-UE: red oak bark extract obtained by ultrasound-assisted extraction with ethanol solvent; ROBE-MA: red oak bark extract obtained by microwave-assisted extraction with water solvent; ROBE-ME: red oak bark extract obtained by microwave-assisted extraction with ethanol solvent.

**Table 5:** Enzyme inhibitory activity of ROBE samples (IC_50_ in μg/mL).

	α-Glucosidase	Tyrosinase	Acetylcholinesterase
ROBE-UA	7.79	ND	2978.00
ROBE-UE	6.94	128.48	1162.00
ROBE-MA	4.92	118.42	1348.00
ROBE-ME	6.21	126.28	863.20
Positive control	Acarbose: 122.27	Kojic acid: 4.44	Galantamine: 1.85 × 10^−4^

Note: ROBE-UA: red oak bark extract obtained by ultrasound-assisted extraction with water solvent; ROBE-UE: red oak bark extract obtained by ultrasound-assisted extraction with ethanol solvent; ROBE-MA: red oak bark extract obtained by microwave-assisted extraction with water solvent; ROBE-ME: red oak bark extract obtained by microwave-assisted extraction with ethanol solvent; ND: not detected.

## Data Availability

Not applicable.
